# Home-based music therapy - a systematic overview of settings and conditions for an innovative service in healthcare

**DOI:** 10.1186/1472-6963-10-291

**Published:** 2010-10-14

**Authors:** Wolfgang Schmid, Thomas Ostermann

**Affiliations:** 1 The German Center for Neurodegenerative Diseases (DZNE), Witten, Germany; 2 Center of Integrative Medicine & Chair of Medical Theory, Integrative and Anthroposophical Medicine, Witten/Herdecke University, Herdecke, Germany

## Abstract

**Background:**

Almost every Western healthcare system is changing to make their services more centered around out-patient care. In particular, long-term or geriatric patients who have been discharged from the hospital often require home-based care and therapy. Therefore, several programs have been developed to continue the therapeutic process and manage the special needs of patients after discharge from hospital. Music therapy has also moved into this field of healthcare service by providing home-based music therapy (HBMT) programs. This article reviews and summarizes the settings and conditions of HBMT for the first time.

**Methods:**

The following databases were used to find articles on home-based music therapy: AMED, CAIRSS, EMBASE, MEDLINE, PsychINFO, and PSYNDEX. The search terms were "home-based music therapy" and "mobile music therapy". Included articles were analyzed with respect to participants as well as conditions and settings of HBMT. Furthermore, the date of publication, main outcomes, and the design and quality of the studies were investigated.

**Results:**

A total of 20 international publications, 11 clinical studies and nine reports from practice, mainly from the United States (n = 8), were finally included in the qualitative synthesis. Six studies had a randomized controlled design and included a total of 507 patients. The vast majority of clients of HBMT are elderly patients living at home and people who need hospice and palliative care. Although settings were heterogeneous, music listening programs played a predominant role with the aim to reduce symptoms like depression and pain, or to improve quality of life and the relationship between patients and caregivers as primary endpoints.

**Conclusions:**

We were able to show that HBMT is an innovative service for future healthcare delivery. It fits with the changing healthcare system and its conditions but also meets the therapeutic needs of the increasing number of elderly and severely impaired people. Apart from music therapists, patients and their families HBMT is also interesting as a blueprint for home based care for other groups of caregivers.

## Background

The use of music as a therapeutic option to support health dates back to ancient times. According to Bailey [1] some of the earliest notable mentions in Western history are found in the writings of ancient Greek philosophers Aristotle and Plato. In contemporary history, Michigan State University offered the first music therapy degree program worldwide in 1944. From that point, music therapy has established itself as a growing health profession for inpatients treatment of psychiatric diseases like schizophrenia or schizophrenia-like illnesses [2], psychosis [3], neurological diseases like multiple sclerosis [4], dementia [5], or for the treatment of patients with chronic pain [6,7].

Due to changes over the last few decades in almost every Western healthcare system towards more outpatient-centred healthcare programs, music therapy also moved into the field of primary care. The first MEDLINE-listed report on music therapy in a day care centre was already published by Pierce et al. [8] in 1964. Since then, music therapy as a discipline has developed its methods and working fields in manifold ways and has also proven to be effective in the field of ambulatory work. For the treatment of chronic diseases, in particular study results for the treatment of tinnitus [9], cardiac rehabilitation patients [10,11], or oncological patients [12], reveal the effectiveness of music therapy also in primary care settings.

Under these circumstances music therapy concepts are of fundamental interest for the continuity of care between in- and outpatient treatment. In particular, severely impaired or geriatric patients discharged from hospital risk losing therapeutic continuity because they are often not able to attend day care centres where music therapy is provided. Therefore, two major directions have been established within the field of music therapy. On the one hand, community music therapy programs focusing on the social and cultural relations of the client and establishing music therapy within his or her social context [13]; on the other hand, music therapy initiatives providing home-based music therapy (HBMT) in manifold projects are of increasing importance [14]. Such HBMT-approaches focus on the perspective of a patient's condition after clinic or rehabilitation and the continuity of the therapeutic process. Specifically for patients after a traumatic brain injury, chronic diseases as well as in palliative and dementia care, a long term treatment concept should include not only functional therapies but also mind-body interventions like music therapy in order to meet the emotional, social, and communicative needs of a patient and to accompany him or her in their coping process [15].

For the first time, this systematic overview focuses on settings and conditions of home-based music therapy programs. The main questions were whether and if so how HBMT can be part of an innovative and effective service for people who have to be treated at home.

## Methods

**Figure 1:**
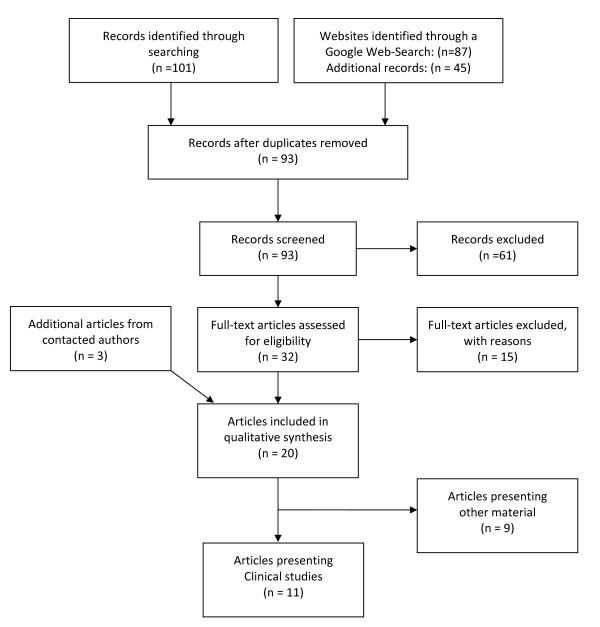
Flowchart of the literature review process

The following electronic databases were used to find articles on home-based music therapy: AMED, CAIRSS, EMBASE, MEDLINE, PsychINFO, and PSYNDEX. Databases were searched from their inception until 2010. The search terms were "home-based music therapy" and "mobile music therapy". In addition, an internet search was performed using Google Scholar. All articles found this way were fully read and their reference lists were checked for further relevant publications. To address a wide range of HBMT, there were no limitations in the initial search in terms of language, year, status, and form of publication. One of the inclusion criteria for all publications was that the music therapy services had to take place in the home of the clients. Therefore, all publications on other out-patient music therapy services were excluded. The complete search strategy together with the number of excluded studies is given in Figure 1.

If a study was found to be eligible, its methodological quality was assessed by two independent reviewers and recorded on a pre-determined checklist together with the basic trial data regarding participants, interventions, settings, outcomes, and study designs (PICOS) [16]. This specifically included quality and duration of MT intervention and appraisal of MT-specific outcomes. Disagreements in data extraction were resolved by consensus. Although blinding of patients in music therapy is not applicable and thus the use of the JADAD score [17] is problematic, the reviewers tried to grade the methodological quality of the studies by the following checklist (inter-rater assessment): adequate description of the study design (retrospective, prospective, multicentre study), subject assembly process (randomization, matched pairs, etc.), comparability of groups, description of drop outs, allocation concealment, description of the intervention, description of statistical analysis, external validity (representative patients, relevant therapeutic concepts, generalization of results).

As the anticipated outcome was assumed to be a mixture of approaches, settings, legal aspects and outcomes relating to the perspectives of the patients, caregivers, and relatives and also due to the expectation of a quite heterogeneous data with respect to study design and used outcome measures, the authors decided against carrying out a meta-analysis of the results and decided to present the findings in a qualitative data synthesis.

## Results

In total, the search identified 93 records after removing duplications. Of those, 61 did not sufficiently cover the field of home-based music therapy or were related to other therapeutic interventions. Thirty-two publications were found to be eligible for full text screening. However, another 15 articles had to be excluded due to reasons of setting while three additional articles, which had not been found by the database search, were retrieved from contacted authors (See Figure 1).

Finally, 20 international publications from the United States (n = 8), Germany (n = 4), Australia (n = 5), Austria (n = 1), Japan (n = 1), and New Zealand (n = 1) were included in the qualitative synthesis (see Tables 1 and 2 for details). From those 20 publications, 11 are clinical studies and nine are reports from practice including field or project reports with single case studies or special music therapy programs for HBMT as well as legal and administrative aspects of HBMT. The 11 studies investigate the effectiveness of HBMT on certain symptoms, mainly on depression, anxiety, distress, and pain [18-21], but also on dyspnea [22], the length and quality of life [23], or the promotion of social skills from autistic children [24,25]. Four studies concentrate on the question of how HBMT could meet not only the needs of the patients, but also the needs of caregivers, spouses, and families [26-29]. One study investigates new strategies to improve therapy outcomes of depression treatment due to the fact that only less than half of the diagnosed patients with depression seem to respond to psychosocial or antidepressant treatment [18].

**Table 1:** Studies in HBMT

Authors	Year	Country	Participants/Conditions	Purpose of the study	Intervention/HBMT Technique	Comparison	Outcomes	Study design
Hanser, SB & Thomson, LW	1994	USA	30 depressed home-bound adults ranged in age from 61 to 86	To examine a music-facilitated psychoeducational strategy as a cost-effective and accessible intervention	8 week program of receptive MT including 8 MT-techniques combined with mind/body interventions	Three groups with 10 patients each: (1) HBMT, (2) phone administered, and (3) control	Both HBMT and self-administered MT significantly increased in tests of depression, distress, self-esteem, and mood and remained stable after 9 month follow up	3-armed RCT with 9-month follow-up
Shiraishi, IM	1997	USA	14 multi-risk mothers ranged in age from 21 to 41 years	To explore the prevalence of anxiety and depression and the effectiveness of MT on these symptoms	8 week program of receptive MT	Three groups: (1) control (n = 4), (2) phone administered (n = 5) and (3) HBMT (n = 5)	Both HBMT and phone administered MT decreased depression and increased self-esteem levels. HBMT had subjective better gains than phone administered mothers	3-armed RCT
McBride, S	1999	USA	24 patients with chronic obstructive pulmonary disease (COPD) (mean age: 69 ± 5,7 years)	To examine the feasibility of using music as an intervention for dyspnea and anxiety	Receptive instrumental MT (classical, new age, easy listening) on an individual schedule	No comparison	Using preferred music as an intervention led to a decline of dyspnea and anxiety	Single armed observational study with repeated measures
Hilliard,, RE	2003	USA	80 adults diagnosed with terminal cancer (mean age: 66 years (MT group) and 65 (control group)	To evaluate the effects of MT on quality of life and length of life in care	Individualized music therapy with a variety of interventions, e.g. singing, listening or instrument playing	Two groups: (1) Routine hospice services and MT versus (2) routine hospice services only	Results clearly support music therapy in hospice and palliative care for improving quality of life of people diagnosed with terminal cancer	Randomized experimental control-group design
Pasiali, V	2004	USA	3 children (7,8 and 9 years old) with autism (diagnosis ranging from high functioning to mildly impaired)	To investigate the effect of presciptive songs on promoting social skills aquisition by autistic children	Prescriptive therapeutic songs	No comparison	Results are not conclusive, but there are some hints that prescriptive songs are a viable intervention for children with autism	Three case studies with ABAB reversal design
Siedlecki, SL	2005	USA	60 patients aged from 21 to 65 with chronic non-malignant pain (CNMP)	To examine the effect of two music-listening interventions on measures of power, pain, depression, and disability in individuals with chronic non-malignant pain	Two music-listening interventions: a standard music (SM) intervention and a patterned music therapy (PM) intervention with pleasant familiar instrumental or vocal music or the sounds of nature	Three groups: (1) standard care without MT, (2) standard music interventions, (3) a patterned music therapy	Both music-listening interventions were equally effective for increasing power, and decreasing pain, depressive symptoms, and disability associated with CNMP. Patients were taught to use music at home to moderate these symptoms	3 armed RCT
Muthesius, D	2005	Germany	40 patients with dementia	To explore the effectiveness of HBMT and the linked support for the caring situation	Singing songs and playing familiar music	No comparison	Similar effects to in-house treatment like emotional support or orientation. More and detailed biographical details from patients, their musical resources support carers and relatives to get positive impressions of the patients	Observational study with nested in single case vignettes
Chiang, JYK	2008	New Zealand	4 carers (three mothers and one speech-langu age therapist) of children with special needs	To investigate how carergivers and other professionals perceive the music therapy process over time	Instrument playing, movement and listening to music, and singing of songs	No comparison	From the perspective of caregivers, music therapy allows them to collaborate with the music therapist. MT contributes to the development of reflective skills for delivering effective professional practice	Explorational qualitative study design with semi-structured interviews
Baker, F et al.	On-going	Australia	120 couples where one partner has a probable diagnosis of dementia	To advance the understanding of MT-techniques to enhance spousal relationship and reduce functional and emotional strain on the spousal caregiver	Singing familiar songs, movement to music, listening to music control: recreational reading intervention	Two groups: (1) active music intervention and (2) control group with recreational reading	Ongoing study	2 armed RCT
Thomas, A, et al.	2009	Australia	191 clients of the Eastern Palliative Care (EPC) from 2007 to 2008	To evaluate the effectiveness of single MT in community based palliative care. Perspectives of clients, carers and therapists are included	Live or recorded music provided by Registered Music Therapists at the EPC who are specifically trained to support people in their own home with music therapy sessions	No comparison	Music therapy supports clients with a life-threatening illness to maintain and/or improve their quality of life and also supports family members in their role as caregivers.	Observational study with nested in qualitative study
Brandes, V	2010	Austria	203 patients with depression and/or burnout and an average age of 49,6 +13,1 years	To investigate new strategies to improve therapy outcomes in psychosocial and antidepressant treatment	Individualized short-term receptive music therapy. MT was administered as single therapy or add-on therapy to antidepressants and/or psychotherapy	Four groups: (1) MT with specific newly composed music, (2) MT with specially arranged classical music, (3) a placebo group receiving nature sounds, and (4) a waiting-list control group.	Individualized short-term music therapy is beneficial as alternative or complementary depression treatment	4 armed RCT

Although cost effectiveness of HBMT is an intrinsic motivation for most of the studies, it is mentioned directly by only one author [19].

To establish a level of quality and understanding of the search and the results of this review, the authors decided to analyze only the 11 clinical studies. With respect to music therapy as a young discipline in health services research and the pioneering character of home-based music therapy work in practice, the nine reports and technical papers are listed in Table 2.

**Table 2:** Reports from HBMT-Practice

Author	Year	Country	Type of publication	Participants/Conditions	Purpose of the publication	Intervention/HBMT-Technique	Outcomes
Oliver, S	1989	USA	Journal article	Developmentally disabled adults and children, acute and chronic psychiatric adults	To report on music therapy services including HBMT	Group and single music therapy	Information related to conceptualizing, initiating, and maintaining a private practice in the music therapy field
Hanser, SB.	1990	USA	Journal article	4 depressed older adults in age from 64 to 75 years	To document and evaluate the implementation of a home based MT-technique	Eight music listening programs in conjunction with known relaxation methods	Four case studies describe successful application of MT to older adults with depression and/or anxiety
Horne-Thompson, A	2003	Australia	Journal article	Patients in palliative care	To compare the role of music therapy within a palliative care facility as opposed to a home	No detailled information	The role of HBMT as opposed to the hospital varies markedly. The necessity to consider new ways to address the needs of patients and their families is emphasized
Weber, S	2003	Germany	Bachelor thesis	Children from underprivilegedfamilies	Review of legal and administrative aspects of HBMT and a single case study	Half year family intervention including active single and group music therapy sessions	HBMT as a new form of socio-paedagogical interventions is successful and cost effective with respect to improvements in family dynamics
Chapman, A.	2004	Australia	Conference talk	Autistic children, a young adult with a physical and intellectual disability and an older woman with dementia	To present practical experiences and considerations on HBMT	Playing instruments, singing songs, vocalizing	Working in the home of a client and his/her family allows current circumstances to be incorporated and enhance this intimacy
Keller, B et al.	2006	Germany	Journal article	Elderly people living at home or in assisted living settings	To report on music therapy services	Multi-methods music therapy	Hints and experiences for music therapists applying HBMT are given
Mager, A	2006	Germany	Journal article	HBMT for elderly, disabled and isolated patients	Position paper	No detailed information	Bringing music therapy to patients in palliative care is considered to be a future task
Roberts, M	2006	Australia	Journal article	Bereaved children and adolescents	To document and compare three types of songwriting in HMBT	Home-based songwriting	Different styles (improvised, computer based, original) of songwriting for HBMT are described and explored
Mirenkov N et al.	2009	Japan	Journal article	Elderly and disabled people living at home	To present and evaluate a concept for applying mobile MT to human-computer interfaces, based on Quality of Life Supporters (QLS) of self-explanatory type, and oriented to elderly and disabled people	A selection of precomposed classical music with different emotional characters	Presentation of a concept for applying mobile music therapy with human-computer interfaces

### Study design

Of the 20 publications, 11 describe outcome studies. Although all studies can be considered small with respect to the number of patients involved, the methodological quality of the studies can be regarded to be quite high. All studies clearly name the outcome parameters, with the majority having a reduction of symptoms like depression or pain or an improvement of quality of life and the relationship between patients and caregivers as primary endpoints. Six studies have a randomized controlled design. The randomized studies include a total of 507 patients, varying form n = 14 to 203 with a mean of 85 participants. A closer analysis of the studies with a modified JADAD score [17] resulted in scores between 1 and 2 due to the impossibility of blinding in music therapy.

### Settings and Interventions

The studies on HBMT show a variety of music therapy interventions and techniques, including active as well as receptive music therapy approaches, and, in one study, Individualized Music-focused Audio Therapies (I-MATs) [18]. Although both active and receptive music therapy can be found in home-based settings, music listening programs play an important role [21,22,27]. In only a few studies, the therapist offered both active and receptive music therapy [24,25].

The musical repertoire offered to the patients contains classical music, new age music or easy listening music like folk or pop songs. Brandes et al. [18] developed and refined two specific listening programs, one with newly composed polyphonic modern music and one with specifically arranged classical music in their four-armed study.

Often the personal preferences of the individual clients are taken into account when choosing the music. According to McBride [22], this is an important variable in producing a relaxation effect. For the music listening programs, the clients get basic technical equipment like cassette players, headphones, as well as tapes with pre-selected music.

Siedlecki [21] offered two music-listening programs in her study: one group listened to a standard music program (SM) and could choose from one of five relaxing instrumental music selections offered. For the second group, patterned music therapy programs (PM) with pleasant familiar instrumental or vocal music or the sounds of nature were offered to a) ease muscle tension or stiffness, b) facilitate sleep or to decrease anxiety, c) improve mood when someone feels sad, angry, or depressed, or d) improve energy level when someone feels fatigued.

The combination of music listening programs with additional therapeutic techniques, like guided imagery, gentle massages, or movement, was quite common [19,27]. Hanser & Thompson [19] presented an eight-step music listening program on the basis of a cognitive-behavioural technique. This program was facilitated especially for use in the home environment and includes slow repetitive music to enhance falling asleep, rhythmic music to enhance energy, and also music listening in conjunction with drawing or painting. McBride [22] relates to this eight-step music listening protocol of Hanser & Thompson in her work and gives her clients, who mainly suffer from chronic obstructive pulmonary disease, precise instructions on the use of music and how to use a music diary to document how their dyspnea and anxiety may have changed after listening to music.

In the field of active music therapy, singing and song writing are very common [24-26]. Pasiali [25] describes a three-step way from listening to singing for her work with autistic children: first she sings the song for the client accompanied by the guitar, then the client joins in and plays rhythmical instruments, like maracas or shaker eggs, to it and finally the client sings the song on his or her own. Chiang [24] offers a 'hello song' at the beginning and a 'good-bye song' at the end of a music therapy session. Activities in her sessions may also involve instrument playing, movement to music, listening, and singing.

In his study on music therapy with people with terminal cancer, Hilliard [23] describes that the techniques of music therapists were singing, lyric analysis, and music-prompted reminiscence as well as the planning of funerals or memorial services.

The information on duration of sessions shows a wide range: most authors reported that the average duration of a session can be between 20 to 60 minutes [18,21,27,30]. The frequency differs from one hour each day for seven days, to twice a day, and from a few times a week to as often as the client wants or needs to have a treatment of music therapy.

### Date of publication

The dates of publication of the articles range from late 1980 to 2010. Four publications from the early years mainly concentrate on conditions and the development of special HBMT-programs, whereas from 2003 onward there is an increasing interest in HBMT as a model of ambulatory service and it is closely related to changes in healthcare systems.

### Fields of applications and patients

Fields of applications of HBMT are similar in all studies: the majority of clients are the elderly living at home and people who need hospice and palliative care [19,21,23,27-29]. For these groups, HBMT is an accessible intervention because often the patients are no longer able to attend a hospital or a therapist's office. Another focus of HBMT is the work within the family-system or with other caregivers or relatives [24-30]. This seems to be a specific and relevant aspect of HBMT, which provides the opportunity for the interaction and integration of family members and caregivers in the music therapy process.

In their study on the treatment of depression, Brandes et al. [18] argues that due to the fact that music therapy is generally not associated with negative side effects, it can be easily implemented with high treatment compliance.

Only the study from Pasiali [25] concentrates on HBMT for children. She investigated how prescriptive songs can promote the acquisition of social skills in autistic children.

### Legal aspects

Naturally, legal aspects of HBMT are very closely linked to the peculiarities of the healthcare system of the specific country. For that reason the reports from out of practice give relevant information on how a music therapist can establish ambulatory work [31-33]. There is an interesting tendency concerning the main focuses of the publications; those from Germany mainly focus on legal aspects and conditions of a HBMT-setting in a changing healthcare system. Keller et al. [33] describe their motivation of establishing HBMT directly linked with the changes in healthcare politics and systems. They developed several ambulatory services initially for homes for the elderly and day care centres to address a new spectrum of clients and paying authorities.

## Discussion

For the first time, this review presents an overview of the field of home-based music therapy and describes settings and conditions represented by an international spectrum of articles.

According to our findings, publications on HBMT cover two main aspects. The first is the search for new fields and forms of work for music therapists alongside with the changes in the health care systems. The second aspect is to meet the challenges of a society with an increasing number of chronically ill patients and people of old age and low mobility and to adapt health care services accordingly.

The publications on HBMT show that receptive music therapy or singing songs with the clients play a predominant role in this working field. This might have organisational as well as economic reasons and takes into account the conditions of the ambulatory setting where the music therapist cannot carry too many instruments with him or her. Also the client as well as his or her relatives can listen to the music whenever it is wanted or needed. Accompaniment with a CD can be helpful when singing familiar or preferred songs. Nevertheless, further development and investigation of music therapy techniques and programs for the specific use in a home-based setting are important for the future of HBMT. Such can be found in publications from Austria, Australia, and the United States.

The article by Horne-Thompson [34] might be helpful for finding out specific aspects of working in the home of a patient. She compares the role of music therapy within palliative care facility, as opposed to home, and looks at the differences of music therapy at a hospital and at a patient's home. Horne-Thompson states that although similar aims are addressed, the differences between hospital and home-based music therapy are striking. The author found that four factors influence the quality of a music therapy service in the home as opposed to the hospital: a) The way in which music therapy is introduced can ultimately determine whether or not a patient will participate, b) The role of the music therapist also varies enormously between a home and a hospital. Whereas in a hospital, music therapists are often perceived by patients as one of the less threatening health professionals, offering pleasant music and instruments, a visit at a patient's home may be experienced as invasive and intrusive due to the large number of health professionals involved in a patient's ambulatory care. c) The session length between home and hospital varies significantly; the direct contact time with a patient in his or her home is more than double the time spent with patients in the hospital. One possible explanation for this observation might be that patients and relatives often plan the daily schedule around the sessions which then results in fewer interruptions. d) One of the main differences is the presence of a spouse or family member while music therapy takes place. This means that they can give important and precise information about the patient's condition and can be involved in the session to address not only the needs of the patient, but also the needs of the family. Horne-Thompson [34] concludes that while family members are often present during sessions, the music therapist treats the family members alongside the patient.

This aspect of HBMT as a kind of "systemic music therapy approach" is the subject of several studies. Thomas et al. [28] found that music therapy in palliative care can assist family members in their role as caregivers while providing quality time with their loved ones.

While spousal caregivers of people with dementia experience the care for their spouse over long periods of time as a significant burden with ever increasing physical, emotional, and social strain, in their ongoing study Baker et al. [27] concentrate on the relationship of persons with dementia and their spousal caregivers. In this study, several specific hypotheses focusing on the caregiver's perceived quality of the spousal relationship as well as the caregiver's satisfaction with the care following the home-based music therapy intervention are tested. The authors assume that the promotion of the caregiver's well-being can keep committed couples living together in their own home and has, therefore, significant economizations. As the study is in progress, there are no results of this very interesting perspective of HBMT at the moment.

In her pilot project, Muthesius [29] investigated how music therapy can support caregivers and caring conditions in the home-based care setting of gerontopsychiatric patients. Muthesius was able to show that for people with dementia HBMT can not only relieve the strain on the care-recipients but also on the caregivers.

Chiang [24] presumes that music therapy in a home environment could help to enhance the child-parent bond. It is also more convenient for the family as HBMT is saving both caregiver and child from travelling to the sessions and it gives opportunities to other family members to be involved and get to know what music therapy is all about. Parents view music therapy as beneficial and enjoyable for their hospitalised child. Parents reported that music therapy could support them in understanding their child's ability to learn through music and how they could use music to interact with their child.

## Limitations of this review

Although we are confident that our literature search with an extensive internet search, hand-searching of grey literature and relevant conference proceedings and communication with experts in the field has identified all relevant papers on HBMT, there might still be unexploited sources of studies we might have missed. However, according to Mc Auley [35], grey literature tends to include small trials with inconclusive results and thus this might not influence the conclusion of this review on the level of outcome. Nevertheless, on the level of implementation of services, valuable information might still be overseen this way. This limitation also holds for excluding publications from Asia. We are aware that the healthcare systems in countries like Japan or China are different than Western healthcare systems; however, it might be interesting to explore how outpatient services are realized under different conditions. In our review, only the paper of Mirenkov et al. [36] gives some insights into HBMT in Japan, mainly from the technical site. However, according to the fact sheet of music therapy in Japan from Ikuno [37], the typical population and the places music therapists work seem to be comparable. In particular, Ikuno points out that music therapy for developmentally disabled children is provided in the home of the client or therapist.

Finally, our internet search discovered a lot of HBMT initiatives in different countries which seem to be implemented in communal healthcare services. Unfortunately, a closer investigation did not reveal any publications about the outcomes of these services. As most of these services are integrated into local healthcare services for a longer time, successful outcomes can be assumed. In total, despite efforts to find all relevant publications, we can only give a fragmented view of the current state of play of HBMT without drawing a representative picture.

## Conclusion

The main questions for this systematic overview were whether HBMT can be part of an innovative and effective service for people who have to be treated at home and, if this is possible, how could it be implemented. It was of particular interest to identify the types of clients and conditions that can be addressed by HBMT and to understand which specialized MT-approaches and settings are needed for a home-based service.

As a result of this review, there are only a few music therapy initiatives in the field of HBMT so far and quite a small number of clinical research publications in this area. Nevertheless, we were able to detect distinct fields of application for HBMT and special techniques for home-based services. The growing interest in HBMT in the last 10 years may indicate the potential of this service and refer to several advantages of HBMT as a future service in healthcare systems. HBMT is interesting for music therapists as well as for special groups of patients and their families and caregivers. This goes alongside the changing healthcare systems and its changing conditions but also it meets therapeutic challenges and needs of an ageing society with an increasing number of elderly and severely impaired people [38,39].

The possibility of integrating spouses, family members, or caregivers in HBMT and instructing them in selected music therapy techniques is an interesting aspect of future HBMT-services in view of lasting and economical aspects. It should be investigated in further studies. We would like to encourage researchers and music therapists to make distinct differentiations of approaches with clear research-based concepts for special groups of patients and to publish their results.

In a more global scope of public health, HBMT could serve as a blueprint for other researchers in the expanding field of home-based care services. Knowing more about the problems and challenges before implementing home-based care services may help to reduce the efforts in structural planning and to improve the quality of psychosocial support. This is not only limited to related interventions like art therapy, but also may hold true for other kinds of individualized mind-body therapies applied to an ever-growing amount of patients who are unable to leave their homes.

## Competing interests

The authors declare that they have no competing interests.

## Authors' contributions

WS and TO both developed the research design for the review. WS was responsible for the literature research and TO screened the grey literature and contacted the corresponding authors. WS was mainly responsible for the data extraction, while TO cross-checked the results. Both WS and TO carried out the qualitative analysis, discussed the results, and wrote the paper.

## Pre-publication history

The pre-publication history for this paper can be accessed here:

http://www.biomedcentral.com/1472-6963/10/291/prepub
